# International comparison of the factors influencing reimbursement of targeted anti-cancer drugs

**DOI:** 10.1186/s12913-014-0595-0

**Published:** 2014-11-29

**Authors:** Carol Sunghye Lim, Yun-Gyoo Lee, Youngil Koh, Dae Seog Heo

**Affiliations:** Seoul National University College of Medicine, Seoul, Korea; Department of Internal Medicine, Kangbuk Samsung Medical Center, Sungkyunkwan University School of Medicine, Seoul, Korea; Department of Internal Medicine, Division of Hematology and Medical Oncology, Seoul National University Hospital, Seoul National University College of Medicine, 101 Daehak-ro, Jongno-gu, Seoul, 110-744 Republic of Korea

**Keywords:** Anti-cancer drugs, Reimbursement, Cost-effectiveness

## Abstract

**Background:**

Reimbursement policies for anti-cancer drugs vary among countries even though they rely on the same clinical evidence. We compared the pattern of publicly funded drug programs and analyzed major factors influencing the differences.

**Methods:**

We investigated reimbursement policies for 19 indications with targeted anti-cancer drugs that are used variably across ten countries. The available incremental cost-effectiveness ratio (ICER) data were retrieved for each indication. Based on the comparison between actual reimbursement decisions and the ICERs, we formulated a reimbursement adequacy index (RAI): calculating the proportion of cost-effective decisions, either reimbursement of cost-effective indications or non-reimbursement of cost-ineffective indications, out of the total number of indications for each country. The relationship between RAI and other indices were analyzed, including governmental dependency on health technology assessment, as well as other parameters for health expenditure. All the data used in this study were gathered from sources publicly available online.

**Results:**

Japan and France were the most likely to reimburse indications (16/19), whereas Sweden and the United Kingdom were the least likely to reimburse them (5/19 and 6/19, respectively). Indications with high cost-effectiveness values were more likely to be reimbursed (ρ = −0.68, *P* = 0.001). The three countries with high RAI scores each had a healthcare system that was financed by general taxation.

**Conclusions:**

Although reimbursement policies for anti-cancer drugs vary among countries, we found a strong correlation of reimbursements for those indications with lower ICERs. Countries with healthcare systems financed by general taxation demonstrated greater cost-effectiveness as evidenced by reimbursement decisions of anti-cancer drugs.

## Background

Each country has limited financial resources devoted to public healthcare; thus, how to best allocate these finite resources in an efficient and justifiable way poses a great challenge at the national level. Especially, expenditure on cancer drugs imposes substantial burdens on patients and providers of health insurance in all countries [1-4]. The costs of cancer drugs have arisen concurrently with the shift from conventional cytotoxic drugs to targeted therapies, including monoclonal antibodies and tyrosine kinase inhibitors, in the application of new drugs for cancer treatment [5,6].

Considering that many new anti-cancer drugs are used to provide modest survival benefits and sometimes solely for the purpose of palliative treatment, cost-effectiveness must be addressed in addition to clinical efficacy [7,8]. For example, Moore *et al*. reported a statistically significant survival benefit of 0.33 months (6.24 *vs.* 5.91 months) for erlotinib plus gemcitabine compared to gemcitabine alone for first-line treatment of advanced pancreatic cancer [9]. However, the result of this finding regarding the value of erlotinib’s small survival benefit has been controversial; the question remains whether we should pay for a drug that improves survival by a median of 10 days with an incremental cost-effectiveness ratio (ICER) of up to $500,000 per year of life saved [8]. To provide guidelines for such kinds of questions, many countries have adopted evidence-based health technology assessment (HTA) programs that analyze the clinical and cost-effectiveness of selected medical technologies to serve as part of the basis for their recommendations [3,10-12]. Using their national assessment programs, France, Germany, and the United Kingdom (UK) have decided not to reimburse erlotinib for first-line treatment of advanced pancreatic cancer, whereas Korea, Japan, and the United States (US) have chosen to reimburse this indication.

As mentioned above, it is noteworthy that even with the same clinical evidence for anti-cancer drugs, reimbursement policies vary among countries [13] because the criteria of cost-effectiveness and additional costs are country-specific and difficult to standardize [14]. The goal of this study was to compare the pattern of publicly funded cancer drug programs in ten countries, and to identify the major factors that explain the differences according to the perspective of each country’s health care system.

## Methods

### Countries and drug indications

For this study, we selected countries that have both universal health care and national or regional public drug reimbursement programs funded by general taxation or premiums. The following ten countries with significant structural and cultural differences in regards to their respective health care system were included: Australia, Canada (Ontario), France, Germany, Japan, Korea, Sweden, Taiwan, the UK, and the US. All countries except Taiwan are the Organization for Economic Co-operation and Development (OECD) member countries; Taiwan was included to better balance the Eastern and Western countries selected for this study. The US was included for comparative purposes, although it has a multi-payer system comprised of private insurance and social insurance programs, with Medicare covering people over 65 years of age and social welfare programs, such as Medicaid, available for low-income people. However, it is not the case for private insurers or indeed for the tens of millions of US citizens that are uninsured. Canada is also unique because drug reimbursement is the responsibility of each province and territory. This study chose to include Ontario to represent Canada, as it is Canada’s most populated province.

In choosing the drugs, we identified molecularly targeted anti-cancer drugs that were approved after 2004 and are variably used in our ten countries of interest. The following thirteen anti-cancer drugs were selected: bevacizumab, cetuximab, crizotinib, dasatinib, erlotinib, imatinib, lapatinib, lenalidomide, nilotinib, pemetrexed, sorafenib, sunitinib, and temsirolimus. We then selected their 19 indications to ensure coverage of a variety of both solid and hematologic malignancies, and mechanisms of action. These indications have evidence of clinical efficacy with modest survival benefit (Table 1) and a resulting ICER in the range of $15,000 ~ $450,000. Herceptin for breast cancer, and rituximab for malignant lymphoma were not included because most countries reimburse both drugs without much controversy. Some new drugs were excluded, because they were available only in selected countries.

**Table 1:** **Reimbursement approval year for indications of 13 anti-cancer drugs in 10 countries (as of February 3, 2013)**

**Drug**	**Indications**	**Countries**									
		**Australia**	**Canada (Ontario)**	**France**	**Germany**	**Japan**	**Korea**	**Sweden**	**Taiwan**	**UK**	**US** ^*^
Bevacizumab	Colorectal cancer : with irinotecan, fluorouracil, and leucovorin	No	2009	2005	2009	2007	No	No	2011	No	2004
	Colorectal cancer : with oxaliplatin, fluorouracil, and folinic acid	2008	2009	2008	2009	2009	No	No	No	No	2006
	NSCLC : 1st line with platinum-based chemotherapy	No	No	2008	2009	2009	No	No	No	No	2006
	Renal cell carcinoma : 1st line with interferon-α	No	No	2008	2009	No	No	No	No	No	2009
Cetuximab	Colorectal cancer : with irinotecan	2010	2011	2009	2009	2008	No	No	2009	No	2004
	Colorectal cancer (*K-RAS* wild type): 1st line with oxaliplatin, fluorouracil, and folinic acid	2010	No	2009	2009	2010	No	No	No	2009	2012
	Head and neck cancer (squamous cell carcinoma) : 1st line with platinum-based chemotherapy	2007	2011	2010	No	No	No	No	2009	No	2011
Crizotinib	NSCLC (*ALK* fusion positive): 2nd line (vs. docetaxel)	No	No	No	No	No	No	No	No	No	2011
Dasatinib	Chronic myeloid leukemia, chronic phase: 1st line	2009	2008	2007	No	2009	2008	2011	2009	No	2010
Erlotinib	Pancreatic cancer : 1st line with gemcitabine	2012	No	No	No	2011	2010	No	No	No	2005
Imatinib	Gastrointestinal stromal tumor: Adjuvant therapy	2011	2008	2009	No	No	2010	2009	2011	no	2008
Lapatinib	Breast cancer with *HER2* overexpression: 2nd line with capecitabine	No	No	2008	No	2009	2010	No	No	No	2007
Lenalidomide	Multiple myeloma : 1st-line	No	2009	No	No	2010	No	2008	No	2009	2006
Nilotinib	Chronic myeloid leukemia, chronic phase: 1st-line	2011	2012	2008	2011	2010	2011	2008	2012	2012	2007
Pemetrexed	NSCLC: Maintenance treatment	No	2008	2008	No	2009	No	No	No	2010	2009
	NSCLC (for non-squamous histology): 1st line with cisplatin	2009	2008	2008	No	2009	2010	No	2009	2009	2008
Sorafenib	Liver cancer : 1st line	2008	2008	2008	2009	2009	2011	No	No	No	2007
Sunitinib	Renal cell carcinoma : 1st line	2010	2008	2006	2009	2008	2007	2006	2010	2009	2006
Temsirolimus	Renal cell carcinoma : 1st-line for poor prognosis patients	No	2011	2008	2009	2010	2011	No	No	No	2007

*Abbreviations:* *UK* United Kingdom, *US* United States, *NSCLC* non-small cell lung cancer.

^*^For United States, Food and Drug Administration (FDA) approval dates are used. However, the fact that FDA approved a certain cancer drug does not mean that that drug is reimbursed.

### Reimbursed indications

The approval years of reimbursement for the 13 anti-cancer drugs were obtained from drug registries of the following authorities of ten countries (as of February 3, 2013): Australia, Pharmaceutical Benefits Scheme in consultation with Pharmaceutical Benefits Advisory Committee (PBAC) [15]; Canada, Ontario Guidelines for Economic Analysis of Pharmaceutical Products in consultation with Ministry of Health and Long-Term Care [16-19]; France, The Haute Autorité de santé in consultation with the Transparency Comission [20]; Germany, Federal Ministry of Health in consultation with the Joint Federal Committee (G-BA) [21] and Institute for Quality & Efficiency in Health Care [22]; Japan, The Ministry of Health, Labour and Welfare in consultation with Chuikyo [23]; Korea, Korea Food & Drug Administration in consultation with Health Insurance Review & Assessment Service [24]; Sweden, Dental and Pharmaceutical Benefits Agency-TLV [25]; Taiwan, Bureau of National Health Insurance in consultation with Drug Beneficiary Committee and Center for Drug Evaluation [26]; the UK, National Health Service in consultation with National Institute for Health and Care Excellence (NICE) [27]; and the US, Food and Drug Administration (FDA) [28]. After identifying insurance coverage data, some of the collected data were then verified by the health care authorities through personal email communications.

The term “reimbursement approval year” refers to the date that the indication was granted reimbursement authorization by each country’s governmental regulatory bodies, such as the HTA agencies. In the case of the US, the date of FDA approval was used. However, it should be noted that FDA approval for a certain cancer drug does not necessarily mean that the drug is reimbursed. The US Centers for Medicare and Medicaid Services typically include all US FDA-approved medications on their formulary, through an evidence-based process without the analysis of cost-effectiveness [29]. On the other hand, private health care plans make formulary decisions individually and these processes vary widely. Because they do not make such information available to the public, it was not possible to find the date of reimbursement that represented the US as a whole.

### Incremental cost-effectiveness ratio

The ICER is commonly used to compare treatments across various indications in cost-effective analysis. The ICER is defined as the ratio of additional costs to incremental benefits of a treatment, and is usually measured as the cost per quality-adjusted life-year (QALY) [30]. The ICER data for each indication were retrieved from the information made available by health authorities online.

### Social health insurance systems

Social health insurance systems of each country were categorized in order to see how they correlated with reimbursement decisions regarding expensive anti-cancer drugs. There are three different types of health care systems based on who collects and provides health care: 1) A multi-payer system comprised of the government-run programs (the dominant insurer) and the private insurances: the government is neither the main healthcare provider nor the collector of money for health care. Individuals have the responsibility to be self-insured; 2) Social health insurance system: the direct provider of health care is not the government, but the government has the financial power to purchase private or public insurance for the people. The fact that the government plays a critical role in the collection of funds for health care does not necessarily mean that the government provides care; and 3) Publicly funded health system: the government directly manages and operates facilities for health care and performs the insurance function of reimbursement. The government runs the single insurance scheme but does not provide the care [31].

For the ten countries of interest, the financing systems were analyzed to determine whether they affected the reimbursement decision of expensive anti-cancer drugs. For each country, the HTA authority was identified and the date of implementation of HTA in the decision making process was also noted. The form of health security was also assessed to categorize countries into different operation systems: customer sovereignty model, social health insurance, and national healthcare service. To estimate the magnitude of expenditure on health care, we also investigated the health expenditure of ten countries using data from the OECD [32].

### Calculation of reimbursement adequacy index

There are two aspects in assessing the reimbursement policy of each country. The first aspect considers how well the policy handles reimbursements for indications that are considered cost-effective. The second aspect considers how well the policy does in not reimbursing indications that are considered cost-ineffective.

In order to assess the cost-effectiveness of reimbursement decisions for the selected anti-cancer drug indications across different countries, we applied a statistical method for measuring the accuracy of diagnostic procedures and calculated the reimbursement adequacy index (RAI) for each country as follows. In case of the US, however, we did not calculate its RAI in that the US has no single dominant health insurance program.

Test outcomes reflect whether an indication is reimbursed or not. To determine the condition status, each indication was assessed to be either cost-effective or cost-ineffective. Although there is no reliable empirical basis on deciding cost-effectiveness thresholds in healthcare, WHO considers that technologies for which the ICER is more than three times a country’s gross domestic product (GDP) per capita will invariably be cost ineffective [6,33]. Therefore, the indications with ICERs less than three times a country’s GDP per capita were considered to be cost-effective in this study.

RAI is the proportion of cost-effective decisions, either reimbursement of cost-effective indications or non-reimbursement of cost-ineffective indications, out of total indications.

$$ RAI\kern0.5em =\frac{\begin{array}{l} number\kern0.5em  of\kern0.5em  reimbursed\kern0.5em  indications\kern0.5em  that\kern0.5em  are\kern0.5em  \cos t\kern0.5em  effective\kern0.5em +\kern0.5em \\ {} number\kern0.5em  of\kern0.5em  not\kern0.5em  reimbursed\kern0.5em  indications\kern0.5em  that\kern0.5em  are\kern0.5em  \cos t\kern0.5em  ineffective\end{array}}{Total\kern0.5em  number\kern0.5em  of\kern0.5em  indications} $$

For example, the cost-effective threshold in Korea was calculated by multiplying Korea’s GDP by 3 ($26881 × 3 = $80643). Therefore, the indications with ICERs less than $80643 were considered to be cost-effective. For Korea, a 2 × 2 table was constructed; the columns represent the condition (cost-effectiveness) and the rows represent the test outcome (reimbursement decision) (Table 2).

**Table 2:** **An example of the calculation of reimbursement adequacy index (RAI) in Korea**

		**Cost-effectiveness**		
		**Effective**	**In-effective**	
Reimbursement	Reimbursed	3	6	9
	Not reimbursed	3	7	10
		6	13	19

$$ \mathrm{R}\mathrm{A}\mathrm{I}\kern0.5em \mathrm{Korea}\kern0.5em =\kern0.5em \frac{3+7}{3+6+3+7}=\frac{10}{19}=0.53 $$

We used Spearman’s rank correlation coefficient for the association between the two variables, and considered *P* <0.05 to be statistically significant.

### Heatmap plot and clustering

To cluster the countries with distinct characteristics of reimbursement policies, we used microarray analysis methods and drew a heatmap plot using MultiExperiment Viwer (MeV) version 4.7. First, we made a 10 × 19 matrix (10 countries and 19 indications) based on Table 1. Then, each indication was sorted from the lowest to highest ICER. For hierarchical clustering, we used a metric algorithm and an average linkage as a linkage method.

### Ethics statement

This study does not involve human subjects, human material, or human data, and therefore, does not require ethics approval. All the data used in this study were gathered from sources publicly available online.

## Results

### Reimbursed indications

Table 1 shows the information regarding whether each country reimburses a certain indication, and if reimbursed, when each country first approved the reimbursement. Not counting the US, Japan and France were the most likely to reimburse the indications (16/19), whereas Sweden and the UK were the least likely to reimburse them (5/19 and 6/19, respectively).

### ICER

**Figure 1:**
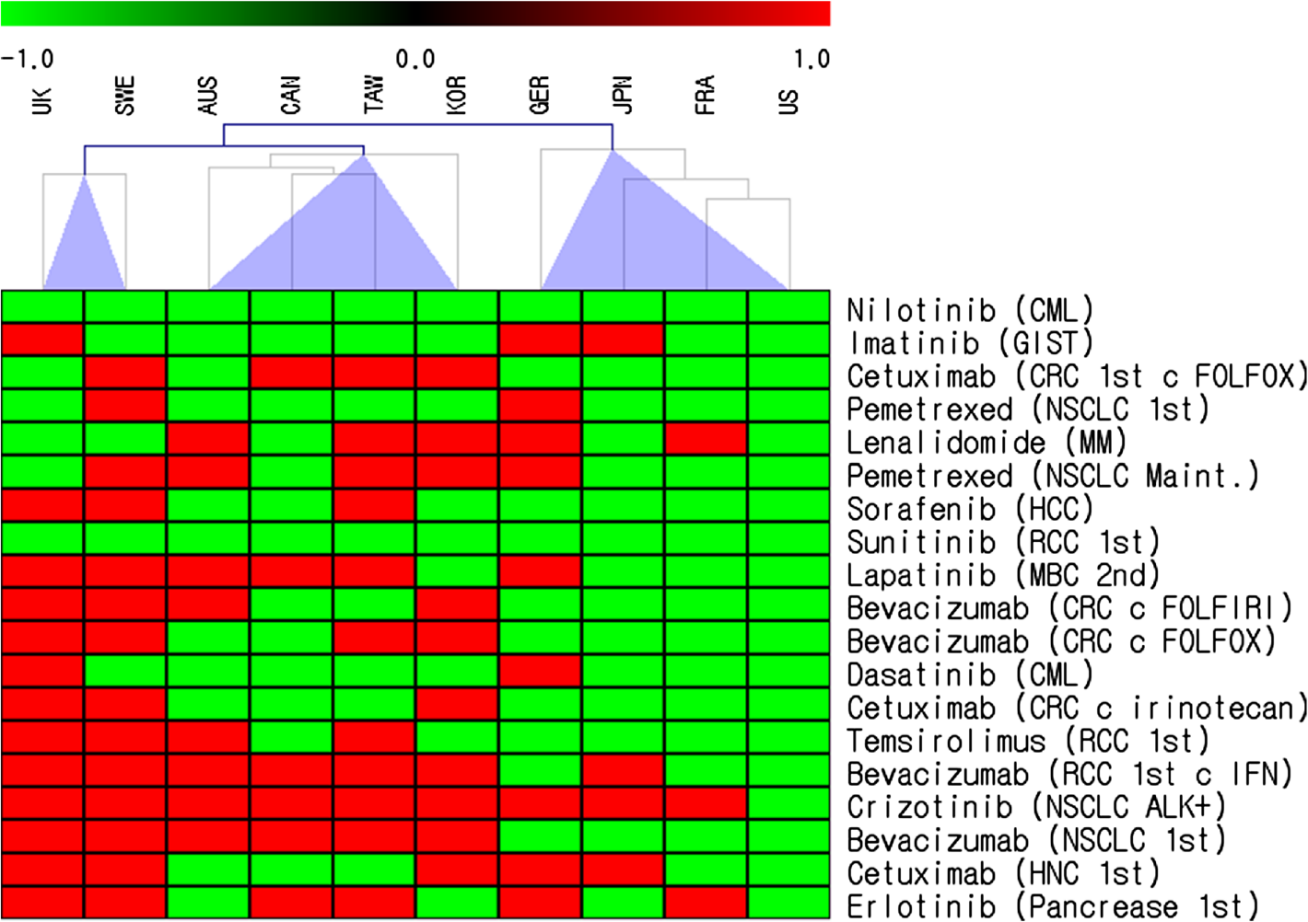
**Clustering of countries according to the pattern of reimbursement status.** The color green is used to show that the indication is reimbursed in a given country, and the color red signifies the indications that are not reimbursed. Each indication is sorted according to its ICER, from the lowest (on top) to highest (on bottom).

The ICER data was available only in two countries: UK (NICE) and Australia (PBAC). We found NICE to be the most comprehensive and used the ICER from NICE as the reference values to use with other countries in which the data was not otherwise available. If the ICER was unavailable due to manufacturer’s unwillingness (bevacizumab plus platinum-based chemotherapy for advanced non-small cell lung cancer) [34] or unissued appraisal (erlotinib plus gemcitabine for advanced pancreatic cancer), we retrieved the relevant ICER value from PBAC (bevacizumab) [35] or through a literature review (erlotinib) [8]. Table 3 shows the representative ICER for each drug indication in the increasing order of ICER values. We found a strong correlation of reimbursements for indications with lower ICERs (ρ = −0.68, *P* = 0.001); the indication with lower ICER was more likely to be reimbursed (Figure 1).

**Table 3:** **Representative incremental cost-effectiveness ratio for each drug indication in increasing order**

**Drug**	**Indications**	**ICER ( in US$)** *
Nilotinib	Chronic myeloid leukemia, chronic phase: 1st-line	17,314
Imatinib	Gastrointestinal stromal tumor: Adjuvant therapy	29,591
Cetuximab	Colorectal cancer (*K-RAS* wild type): 1st line with oxaliplatin, fluorouracil, and folinic acid	42,026
Pemetrexed	NSCLC (for non-squamous histology): 1st line with cisplatin	44,451
Lenalidomide	Multiple myeloma: 1st-line	68,941
Pemetrexed	NSCLC: Maintenance treatment	73,978
Sorafenib	Liver cancer: 1st line	82,792
Sunitinib	Renal cell carcinoma: 1st line	85,572
Lapatinib	Breast cancer with *HER2* overexpression: 2nd line with capecitabine	93,496
Bevacizumab	Colorectal cancer: with irinotecan, fluorouracil, and leucovorin	98,937
Bevacizumab	Colorectal cancer: with oxaliplatin, fluorouracil, and folinic acid	110,967
Dasatinib	Chronic myeloid leukemia, chronic phase: 1st-line	118,050
Cetuximab	Colorectal cancer: with irinotecan	121,528
Temsirolimus	Renal cell carcinoma: 1st-line for poor prognosis patients	128,595
Bevacizumab	Renal cell carcinoma: 1st line with interferon-α	131,070
Crizotinib	NSCLC (*ALK* fusion positive): 2nd line (vs. docetaxel)	158,133
Bevacizumab	NSCLC: 1st line with platinum-based chemotherapy	196,000^†^
Cetuximab	Head and neck cancer (squamous cell carcinoma) : 1st line with platinum-based chemotherapy	261,767
Erlotinib	Pancreatic cancer: 1st line with gemcitabine	430,000^‡^

ICER, Incremental Cost Effectiveness Ratio; NSCLC, non-small cell lung cancer.

*Most ICER values except two indications were retrieved from the National Institute for Health and Clinical Excellence in United Kingdom (costs are expressed in US dollars; **£**1 equals $1.574 (as of January 29, 2013)).

^†^This value was from the Pharmaceutical Benefits Advisory Committee in Australia. (Canadian $1 equals US $0.98) http://www.pbs.gov.au/info/industry/listing/elements/pbac-meetings/psd/2011-03/pbac-psd-bevacizumab-march11.

^‡^This value was from Miksad RA *et al. J Clin Oncol* 25:4506–7; author reply 4508, 2007.

### Social health insurance systems and the reimbursement adequacy index

Table 4 shows the RAI and social health insurance systems across the ten countries. The UK had the highest RAI scores. Four countries with high RAI scores (UK, Canada, Australia, and Sweden) have health care systems that are all financed mainly by general taxation. On the other hand, the three countries with the lowest RAI scores were Korea, Taiwan, and Germany. In these countries, the social health insurance is financed by payroll tax or premiums rather than general taxation. Interestingly, countries with high RAI scores (UK, Canada, Australia, and Sweden) have incorporated HTA since the 1990’s, whereas countries with low RAI scores (Korea, Taiwan, and Germany) have only recently incorporated HTA. With regard to Germany, the G-BA is the supreme decision-making body; however, the G-BA does not conduct economic evaluations requiring an ICER for informing coverage decisions except for those few cases where there is a disagreement between the company and the insurer, the G-BA. Similar to Germany, France has a HTA agency that applies an evaluation process based on evidence, not comparative cost effectiveness.

**Table 4:** **Reimbursement adequacy index and social health insurance system**

	**UK**	**Canada**	**Australia**	**Sweden**	**France** ^*^	**Japan**	**Korea**	**Taiwan**	**Germany** ^*^
Reimbursement adequacy index	0.79	0.68	0.63	0.58	0.58	0.58	0.53	0.53	0.47
Financing system	76% General tax +18% National insurance +3% user charge	100% General tax	98.5% General tax +1.5% Levy	100% General tax	33% General tax +43% National insurance + Levies + other social security	Premium	Premium	Premium	Premium
Health technology assessment (HTA)	Government independent	Government independent	Government agency	Government agency	Government agency	N/A	Government agency	Government agency	Government independent
Date of HTA foundation	1999	1990	1993	1987	2004	N/A	2009	2007	2004
Form of Health Security	National Healthcare Service	Social Health Insurance	Social Health Insurance	National Healthcare Service	Social Health Insurance	Social Health Insurance	Social Health Insurance	Social Health Insurance	Social Health Insurance

N/A, not available.

*Germany and France have the HTA agencies that apply an evaluation process based on evidence but not based on comparative cost effectiveness.

**Figure 2:**
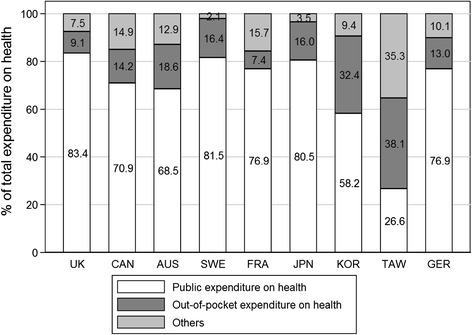
**Proportion of total expenditure on health.** Countries are arranged according to their respective reimbursement adequacy index score (higher scores on the left and lower scores on the right).

We found a significant correlation in which the RAI decreased as the proportion of pharmaceutical expenditure among total health care expenditure increased (ρ = −0.65, *P* = 0.04) (Table 5) (Figure 2). The simple price of the drug (*P* = 0.24), GDP (*P* = 0.19), and the proportion of healthcare expenditure among GDP (*P* = 0.81) did not relate to RAI significantly. Regarding the pattern of reimbursement status (Figure 1), we tried to group countries into those with similar patterns: A) UK and Sweden; B) Australia, Canada, Taiwan, and Korea; C) Germany, Japan, France, and the US. However, we did not find any correlation between clustering results and other indices including the RAI, the date of implementation of HTA systems, and other parameters in Table 5 (unpublished data).

**Table 5:** **Health expenditure of each country**

	**UK**	**Canada**	**Australia**	**Sweden**	**France**	**Japan**	**Korea**	**Taiwan**	**Germany**
GDP per capita in US$ (2009)	34397	37773	39833	37255	33785	32061	26881	32214^*^	35643
Total expenditure on health (TEH), % of GDP (2009)	9.8	11.4	9.1	9.9	11.7	9.5	6.9	6.4^*^	11.7
Public expenditure on health, % of TEH (2009)	83.4	70.9	68.5	81.5	76.9	80.5	58.2	26.6^*^	76.9
Out-of-pocket expenditure (households) on health, % of TEH (2009)	9.1	14.2	18.6	16.4	7.4	16	32.4	38.1^*^	13
Pharmaceutical expenditure and other medical non-durables, % of TEH (2009)	11.8^*^	17	14.7	12.7	16.1	20.8	22.5	25^*^	14.9
Life expectancy of both sexes at birth in years (2009)	80.4	80.8^*^	81.6	81.4	81.1	83	80.4	79^*^	80.3

*Abbreviations:* *GDP* gross domestic product, *UK* United Kingdom, *US* United States.

Source: OECD website (except Taiwan) from http://stats.oecd.org/Index.aspx?DataSetCode=SHA.

For Taiwan, refers to  2012, Vol.3 , No1.

^*^When the data for 2009 was not available, the relevant data from 2008 was used.

## Discussion

The present study shows that reimbursement policies for anti-cancer drugs vary among countries even though they rely on the same clinical evidence of those drugs. Although most counties have adopted evidence-based HTA programs to estimate the cost-effectiveness of anti-cancer drugs, each HTA suggests different recommendations. Overall, there was a strong correlation of reimbursements in indications with lower ICERs.

Among the many factors that might influence reimbursement policies, we focused on ICER, a surrogate index for cost-effectiveness, because the ICER is regarded as well-established evidence for basing resource allocation decisions [30]. As healthcare costs continue to rise, recent clinical trials have tried to integrate ICER into their methods to provide further evidence of potential benefits along with clinical efficacy [36]. Therefore, the decision to place emphasis on ICER seemed to be a rational approach for analyzing the diverse patterns of reimbursement decisions.

We tried to collect ICER data from each country and compare them, but most countries did not disclose reimbursement policies and related data. The UK, Australia, Canada, and Sweden apply Cost-Effectiveness criteria in a fairly rigorous way; on the other hand, France and Germany measure the added clinical value on a 5-point scale, which is then used as a guide for price negotiations. France and Germany do not calculate the ICER at all, and in this sense, the HTA agencies in France and Germany do not conduct economic evaluations for informing reimbursement. The UK and Australia do the most thorough job of establishing an ICER database online. Thus, ICER data from NICE (UK) or PBAC (Australia) were retrieved. In our study, the representative ICER data from NICE were used as the standard for comparison instead of using ICER data from different sources. However, the ICER estimates might vary between countries depending on which current practice is being used as a control, and on assumptions about survival estimates for a new regimen. In addition, utilities for estimating incremental health gain have national differences in their weighting [37]. Thus, other determinants such as controls, real world effectiveness estimates, unit costs, resource use patterns, patient preferences, and the threshold might account for differences in the ICERs and each country’s respective reimbursement decisions. For the reasons mentioned above, there are inherent limitations to extrapolating NICE’s ICER values [6]. Furthermore, because the RAI was calculated based on the ICERs mainly retrieved from NICE (UK), the ranking reflects how well each country adheres to NICE’s method of evaluating cost-effectiveness. By using this standard, there is an inherent assumption that the UK does the best in making cost-effectiveness decisions and at the same time, the UK inevitably obtains the highest RAI according to our method. This is by far the greatest weakness of the analysis.

We found that indications with high cost-effectiveness values (lower ICERs) were more likely to be reimbursed, which suggests that each country considers cost-effectiveness in addition to clinical efficacy when deciding the reimbursement of a certain drug. Given the finite financial resources and rising costs of anti-cancer drugs, each country must be economical when deciding reimbursement policies for each anti-cancer drug. Cheema *et al.* documented international variability in the ability to access cancer drugs by measuring the number of licensed indications reimbursed by public payers [13]. However, they did not go so far as to compare the pattern of reimbursement decisions using a mathematical algorithm to systematically analyze a country’s efficiency in making cost-effective coverage decisions with regard to ICER. Although the findings of our study may have been expected, our study attempts to compare reimbursement policies of different countries with regard to cost-effectiveness by using a systematic approach of calculating RAI.

We tried to cluster the countries according to the pattern of reimbursement using microarray analysis methods (Figure 1). However, we could not find any correlation between clustering results and other indices, including the RAI, government dependency on HTA, and other parameters. This suggests that the reimbursement decision derived from estimates of cost-effectiveness cannot be explained by one index alone.

There are many other factors that influence the decision of authorities to fund certain drugs besides the ICER: the patient’s demographics (i.e. drugs with higher ICERs tend to be more frequently approved for pediatric patients with cancer), the availability of alternative treatments, and the consideration of new drugs with its impact on the jurisdiction’s healthcare budget. Undoubtedly, the decision to approve a new drug is multifaceted and multifactorial. Nevertheless, our findings show that the cost-effectiveness is taken into prime consideration in many countries (especially countries which have adopted HTA since the 1990’s).

We introduced the concept of RAI to estimate how efficiently each country decides to reimburse anti-cancer drugs. Our study found an interesting relationship between RAI scores and social health insurance systems. Specifically, the four countries that had high RAI scores, UK, Canada, Australia, and Sweden have health systems that are financed by general taxation. Additionally, countries with health care systems that are financed by general taxation have operated HTA longer than those that are financed by premium. This shows that countries with a health system financed by general taxation are more likely to consider the cost-effectiveness of certain anti-cancer drugs. Considering the fact that no health insurance system can cover every medical intervention despite the clinical benefit, our findings reflect the realities of current health systems.

Faden *et al*., who had a similar point of view, compared the UK and US experiences with expensive cancer drugs, and found that the UK system is fairer and better structured than the US system when dealing with difficult decisions about expensive end-of-life cancer drugs [38]. They emphasized the role of NICE when facing dilemmas for competing clinical or cost-effectiveness of cancer drugs, which supports our findings.

Our study had other limitations. First, the reimbursement status may not be representative of real-world situations. Some cancer drugs are supported by other sources of funding regardless of HTA recommendations. For example, the Cancer Drug Fund in the UK supports some indications that go against the policies by NICE, although these are not national decisions. Second, the selection of study countries and drug indications are inherent to selection bias. Given the vast number of novel molecular targeted anti-cancer drugs available in the market of the ten countries, it was challenging to select drugs and their indications in a systemic way with clear selection criteria. Furthermore, because the reimbursement decision and the year of reimbursement were found using internet searches, it was very challenging to find reliable information for the purpose of our study. Third, the representative ICER we presented may not perfectly reflect the current appraisal of each country because of the aforementioned reasons. Lastly, the threshold determined to be cost-effective (ICERs being less than three times a country’s GDP per capita) is arbitrary to use as a measure of cost effectiveness irrespective of the country’s health expenditure as a proportion of GDP; for example, the presence of a functional generics market would increase the productivity of the system with regard to branded drugs. Recent empirical research in the UK showed that the threshold is likely to be half the country’s GDP per capita [39]. However, despite these limitations, we tried to get the gist using the best available evidence.

## Conclusion

Although reimbursement policies for anti-cancer drugs vary among countries, we found a strong correlation of reimbursements favoring indications with lower ICERs. Countries that have health systems financed by general taxation adopt cost-effectiveness as evidenced by healthcare resource allocation.
